# The Bio‐Hermes Study: Biomarker database developed to investigate blood‐based and digital biomarkers in community‐based, diverse populations clinically screened for Alzheimer's disease

**DOI:** 10.1002/alz.13722

**Published:** 2024-02-28

**Authors:** Richard C. Mohs, Douglas Beauregard, John Dwyer, Jennifer Gaudioso, Jason Bork, Tamiko MaGee‐Rodgers, Mickeal N. Key, Diana R. Kerwin, Lynn Hughes, Cyndy B. Cordell, The Bio‐Hermes Collaborative Group, Jessie Nicodemus‐Johnson, Craig Mallinckrodt, Robin Wolz, Kevin Yarasheski, Joel B. Braunstein, Tim West, Philip Verghese, Kris Kirmess, Matthew Meyer, David Wilson

**Affiliations:** ^1^ Global Alzheimer's Platform Foundation Washington District of Columbia USA; ^2^ Kerwin Medical Center Dallas Texas USA; ^3^ Advisor to the Global Alzheimer's Platform Foundation and IXICO plc London UK; ^4^ Advisor to the Global Alzheimer's Platform Foundation Washington District of Columbia USA

**Keywords:** Alzheimer's disease, Alzheimer's disease blood‐based biomarkers, Alzheimer's disease ethnic and racial differences, amyloid beta 40, amyloid beta 42, amyloid beta 42/40, Bio‐Hermes Study, clinical trials, phosphorylated tau181, phosphorylated tau217, screening

## Abstract

**INTRODUCTION:**

Alzheimer's disease (AD) trial participants are often screened for eligibility by brain amyloid positron emission tomography/cerebrospinal fluid (PET/CSF), which is inefficient as many are not amyloid positive. Use of blood‐based biomarkers may reduce screen failures.

**METHODS:**

We recruited 755 non‐Hispanic White, 115 Hispanic, 112 non‐Hispanic Black, and 19 other minority participants across groups of cognitively normal (*n* = 417), mild cognitive impairment (*n* = 312), or mild AD (*n* = 272) participants. Plasma amyloid beta (Aβ)40, Aβ42, Aβ42/Aβ40, total tau, phosphorylated tau (p‐tau)181, and p‐tau217 were measured; amyloid PET/CSF (*n* = 956) determined amyloid positivity. Clinical, blood biomarker, and ethnicity/race differences associated with amyloid status were evaluated.

**RESULTS:**

Greater impairment, older age, and carrying an apolipoprotein E (apoE) ε4 allele were associated with greater amyloid burden. Areas under the receiver operating characteristic curve for amyloid status of plasma Aβ42/Aβ40, p‐tau181, and p‐tau217 with amyloid positivity were ≥ 0.7117 for all ethnoracial groups (p‐tau217, ≥0.8128). Age and apoE ε4 adjustments and imputation of biomarker values outside limit of quantitation provided small improvement in predictive power.

**DISCUSSION:**

Blood‐based biomarkers are highly associated with amyloid PET/CSF results in diverse populations enrolled at clinical trial sites.

**Highlights:**

- Amyloid beta (Aβ)42/Aβ40, phosphorylated tau (p‐tau)181, and p‐tau 217 blood‐based biomarkers predicted brain amyloid positivity.
- P‐tau 217 was the strongest predictor of brain amyloid positivity.
- Biomarkers from diverse ethnic, racial, and clinical cohorts predicted brain amyloid positivity.
- Community‐based populations have similar Alzheimer's disease (AD) biomarker levels as other populations.
- A prescreen process with blood‐based assays may reduce the number of AD trial screen failures.

## 1 BACKGROUND

Accurate and expeditious detection of Alzheimer's disease (AD) pathology continues to be a major hurdle in advancing AD‐modifying clinical research.^1^, ^2^, ^3^ A robust screening process that can identify patients with a high probability to randomize into AD therapeutic research trials would greatly enhance the ability to conduct and reduce the time needed to complete clinical trials.

AD is characterized by the accumulation of two protein aggregates in the brain: extracellular deposits of amyloid beta (Aβ)–containing plaques and intraneuronal aggregates of misfolded tau protein.^4^ Amyloid tracers are now available to detect the presence of amyloid plaques in the brain during a positron emission tomography (PET) scan.^5^, ^6^ Additionally, it has been shown that Aβ levels in cerebrospinal fluid (CSF) can be a valid reflection of brain amyloid deposits.^7^

Numerous AD clinical trials, particularly those targeting either Aβ or amyloid plaques have used amyloid PET scans and/or CSF measures as an inclusion criterion for enrollment. While amyloid PET tracers have been shown to be very accurate in detecting brain amyloid deposits, these scans are costly and impose a significant patient burden. Blood‐based measures that are associated with the presence of brain amyloid plaques have recently been developed.^8^, ^9^, ^10^ Additionally, there is substantial interest in blood‐based biomarkers reflecting two other critical aspects of AD pathology: tau tangles and neurodegeneration. The amyloid/tau/neurodegeneration (A/T/N) framework has been proposed as goal for developing biomarkers of each of these three aspects of AD pathology.^2^

Several clinical studies have been conducted evaluating the ability of various blood‐based biomarkers to identify AD. These studies have identified Aβ40, Aβ42, the Aβ42/Aβ40 ratio (Aβ42/Aβ40), tau, and several species of phosphorylated tau (p‐tau) as good candidates.^8^, ^9^, ^11^, ^12^ Many of the studies investigating the clinical utility of these biomarkers have used clinical groups identified for other purposes, such as autopsy studies,^13^, ^14^ genetic studies,^15^, ^16^ or epidemiological studies.^17^, ^18^ While informative, these studies may not adequately reflect the kinds of patients typically enrolled in therapeutic studies conducted at clinical trial sites and often do not have the racial and ethnic diversity characteristics of the US population.^19^ Data from the Centers for Disease Control (CDC) indicates that the prevalence of AD is higher among non‐Hispanic Blacks and Hispanics than among non‐Hispanic Whites in the United States^20^; at the same time, data from the IDEAS study^21^ indicate that the rate of amyloid‐positive PET scans is lower among non‐Hispanic Blacks and Hispanics than among non‐Hispanic Whites in the United States. Thus, any evaluation of biomarkers should assess whether there are differences by race and ethnicity.

The primary objective of the Bio‐Hermes Study was to evaluate the ability of several promising blood‐based and digital biomarkers to reflect the presence of brain amyloid in participants enrolled at clinical trial sites using recruitment procedures similar to those used in AD therapeutic drug studies. Participants in the Bio‐Hermes Study had clinical characteristics similar to those enrolled in clinical trials of disease‐modifying treatments and, because multiple biomarkers were obtained, the predictive value of biomarkers alone or in combination can be evaluated. The data collected in this study should facilitate the use of biomarkers as prescreening tools to identify trial participants likely to have amyloid deposits in the brain as measured by PET or CSF, thus avoiding time consuming and costly screen failures.

The study also aimed to include a significant representation of racial and ethnic minority participants. While blood samples and digital data were obtained to allow assessment of many biomarkers, this current report presents data on the most used blood biomarkers, Aβ40, Aβ42, Aβ42/Aβ40, total tau (t‐tau), p‐tau181, and p‐tau217. For the great majority of participants, brain amyloid was assessed with amyloid PET; at one site where PET was not available, CSF was analyzed.

## 2 METHODS

### 2.1 Participants and study flow

From April 2021 through November 2022, 17 research sites recruited 1296 study participants from their community‐based populations and were able to enroll 1001 into the Bio‐Hermes Study. All these sites recruit for clinical trials investigating potential new drug treatments for AD and have extensive experience in recruiting participants who are cognitively normal (CN), those with mild cognitive impairment (MCI), and those with mild AD. All sites are also experienced with protocols that require brain amyloid positivity for study enrollment.

**FIGURE 1 alz13722-fig-0001:**
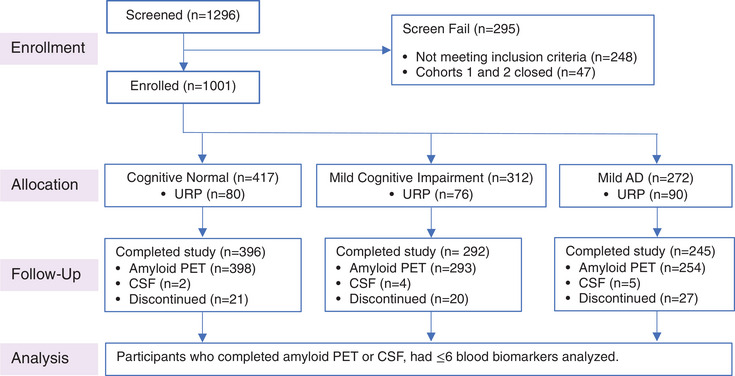
Bio‐Hermes Study CONSORT diagram. AD, Alzheimer's disease; CONSORT, Consolidated Standards of Reporting Trials; CSF, cerebrospinal fluid; PET, positron emission tomography; URP, underrepresented populations.

To ensure appropriate participant stratification into three cohorts and sufficient inclusion of underrepresented populations (URP), recruitment was competitive and closely monitored by the study sponsor. Stratification was based on clinical screening procedures that determined clinical presentation of CN, MCI, or mild AD. Figure 1 shows the Consolidated Standards of Reporting Trials (CONSORT) diagram for the Bio‐Hermes Study detailing the total number of potential participants screened, the number enrolled into each of the clinical cohorts, and the number from traditionally URP. Resources provided by the Global Alzheimer's Platform Foundation (GAP) to facilitate recruitment of minority participants included Community Connectors, mobile screening units, and Inclusive Research Initiative (IRI) Advisory committees. Community Connectors are GAP staff who aid sites in establishing ongoing relationships with minority‐led community organizations; mobile units are large vans equipped with testing and blood drawing facilities that can travel to screenings in areas largely populated by minority groups; and IRI Advisory committees are comprised of leaders from minority communities who meet regularly with GAP and site staff to learn about GAP research studies and provide guidance on strategies for recruitment. Along with these recruitment efforts, sites were required to recruit a percentage of minority group participants to maintain their participation as active study sites.

**RESEARCH IN CONTEXT**

1. **Systematic review**: Blood‐based biomarkers’ reported relationships to brain amyloid have varying estimates of predictive ability in autopsy, genetic, and epidemiological studies. We enrolled community‐based participants who were cognitively normal, had mild cognitive impairment, or mild Alzheimer's disease and compared blood‐based biomarkers to brain amyloid determined by imaging/cerebrospinal fluid studies.
2. **Interpretation**: Non‐Hispanic Whites differed from non‐Hispanic Black and Hispanic participants on some demographic and biomarker values. Strong relationships in clinical, race, and ethnic groups were shown for amyloid beta (Aβ)42/Aβ40, phosphorylated tau (p‐tau)181, and p‐tau217 (strongest) to amyloid positivity. Adjustment for age and apolipoprotein E ε4 carrier status slightly improved predictive power.
3. **Future directions**: These results provide precise estimates of the ability of blood‐based biomarkers to predict brain amyloid positivity in a community‐based population. The predictive power of biomarkers was good in all groups, but demographic and biomarker differences associated with ethnicity and race may influence screen failure rates in trial recruitment and should be further investigated.

### 2.2 Screening procedures and defined cohorts

Participants who met eligibility criteria were further identified as belonging to one of the three clinical cohorts representing the range of cognitive function typically included in therapeutic or prevention trials, specifically CN, MCI, or mild AD. Eligibility criteria included being 60 to 85 years of age (inclusive) and being fluent in language of tests used by the site. English language–based tests were approved for all sites and eight sites were also approved to use Spanish language–based tests. Eligible participants had a Mini‐Mental State Examination (MMSE) score of 20 to 30 inclusive except for a few instances in which the principal investigator (PI) was allowed to include MMSE scores as low as 17 if the patient was determined to have mild AD based on their clinical judgement. Participants had a normal Geriatric Depression Scale (GDS) score,^22^ no known negative brain amyloid PET scan within the past 12 months, no history of stroke or seizures within 12 months, and no history of cancer within the past 5 years (excluding melanoma skin or prostate cancer in situ). Blood biomarkers were not used for screening or for cohort assignment. Excluded from study participation were those with unstable medical conditions that could affect cognitive assessments or who had other conditions that would rule out participation in a therapeutic trial.

The study sought to enroll 1000 participants to evaluate the relationship of biomarkers with brain amyloid status in each clinical cohort; a larger number (400) was sought for the CN cohort due to the expected low rate of amyloid positivity in this group. Cohort status was not shared with participants.

#### 2.2.1 Cohort 1: Cognitively normal

Participants stratified as CN had no self‐reported, or partner‐reported, memory loss or concerns,[Fig alz13722-fig-0001] in addition to the following screening results: MMSE score of 26 to 30 inclusive; Rey Auditory Verbal Learning Test (RAVLT) delayed recall score^23^ within normal range based on age‐ and race‐adjusted means^24^; and in the investigator's judgment, no evidence of functional decline based on the Functional Activities Questionnaire (FAQ) score/study partner report (the equivalent of a score of 0–8).^25^ Potential participants with exceptionally good memory scores on the RAVLT (> 1.5 standard deviation [SD] above age‐ and race‐adjusted mean) were excluded from the study because of the very low likelihood of amyloid positivity in this group. A similar screening procedure was used in the A4 study^3^ because of the great expense involved in completing brain amyloid PET scans on people with a very low likelihood of entering a therapeutic trial.

#### 2.2.2 Cohort 2: Mild cognitive impairment

Participants stratified to the MCI cohort met the following criteria: a diagnosis of MCI based on the National Institute on Aging (NIA)‐Alzheimer's Association (AA) criteria^2^ and verified through medical records, OR had screening results as follows: MMSE score of 24 to 30 inclusive; RAVLT delayed recall score of at least 1 SD below the age‐ and race‐adjusted mean; and in the investigator's judgment, minimal to mild functional impairment but with preservation of independence in functional abilities based on the FAQ score/study partner report.

#### 2.2.3 Cohort 3: Mild AD

Participants in the mild AD cohort met the following criteria: a diagnosis of probable AD based on the NIA‐AA criteria^2^ and verified through medical records, OR had screening results as follows: MMSE score of 20 to 24 except for a few instances in which the PI was allowed to include MMSE scores as low as 17 based on clinical judgement; RAVLT delayed recall score ≥ 1 SD below the age‐ and race‐adjusted mean; and in the investigator's judgment, evidence of functional decline and dependence in functional abilities based on FAQ score/study partner report.

### 2.3 Study visits

Prior to any study procedures, an informed consent was obtained from all study participants. The protocol was reviewed and approved by Advarra, a central institutional review board (Reference Number Pro00046018). The study is registered on ClinicalTrials.gov NCT04733989.

#### 2.3.1 Visit 1 (cognitive testing and blood sampling)

During Visit 1, contact and demographic information were collected, vital signs, height, medical history (including known family history of AD), and concomitant medications were recorded. Cognitive assessments were completed in the following order: MMSE, RAVLT with delayed recall, FAQ, and the GDS. The FAQ and GDS could be administered during the RAVLT test between the initial learning trials and the delayed recall period. (Other cognitive assessments were conducted after the GDS during Visit 1; however, results will be presented in future publications.) Blood samples for biomarker analyses and required lab tests were also collected during Visit 1.

#### 2.3.2 Visit 2 (imaging or CSF collection)

At Visit 2 participants received a brain amyloid PET scan at a designated imaging center OR if the site did not have local access to a qualified PET imaging center, a lumbar puncture for CSF collection was performed.

#### 2.3.3 Visit 3 and follow‐up (review of brain amyloid PET/CSF results and blood collection)

During Visit 3, additional blood samples were collected from participants. Participants also met with their site PI or other appropriate study staff to review their brain amyloid assessment results (unless the participant declined this disclosure). For all participants with brain amyloid positive results, an additional contact occurred within 24 to 72 hours postdisclosure by telephone or via a virtual visit. This follow‐up contact evaluated the participant's overall well‐being using the GDS and provided any warranted referral.

### 2.4 Blood biospecimen collection and management

#### 2.4.1 Blood biospecimen collection

At Visit 1, whole peripheral blood samples were drawn via vacutainer tubes appropriate for subsequent laboratory analyses. For genetic testing, samples were collected in two PAXgene collection tubes and tubes were placed directly into a cyrobox and frozen (−80°C) within 4 hours of the blood draw. Additional blood samples were collected in five K2 ethylenediaminetetraacetic acid (EDTA) tubes. From one K2 EDTA tube, 2.0 mL of whole blood was transferred to conical tubes prior to centrifugation. All five K2 EDTA tubes were centrifuged at a relative centrifugal force of 1500 (±100) x *g*  at room temperature for at least 15 minutes. Postcentrifugation, plasma samples were placed in Simport tubes for transfer. Both whole blood and plasma samples obtained from the K2 EDTA tubes were processed and frozen (−80°C) within 4 hours of the blood draw. At Visit 3, peripheral blood samples were drawn via five K2 EDTA vacutainer tubes and were centrifuged at a relative centrifugal force of 1500 (±100) x *g* at room temperature for at least 15 minutes. Postcentrifuge, plasma samples were placed in Simport tubes for transfer.

The amount of blood collected at each study visit was limited to ≤50 mL. All blood samples were deidentified and all specimens remained at −80°C prior to shipping. Specimens could be stored at −20°C for a maximum of 48 hours before transferring to a −80°C freezer.

Deidentified frozen blood samples were sent to Clinical Laboratory Improvement Amendments (CLIA)–certified laboratories for a lipid panel analysis and analysis of biomarkers of interest for the Bio‐Hermes Study, including Aβ40, Aβ42, Aβ42/Aβ40, p‐tau181, p‐tau217, apolipoprotein E (apoE) ε4, and others commonly associated with AD. Specifically, plasma samples were sent to the following: C_2_N Diagnostics laboratories for Aβ40, Aβ42, Aβ42/Aβ40, and apoE ε4 (PrecivityAD) assays^26^, ^27^; Quanterix laboratories for Simoa analysis of Aβ40, Aβ42, Aβ42/Aβ40, neurofilament light (NfL), glial fibrillary acidic protein (GFAP), t‐tau, and p‐tau181^28^; Eli Lilly and Company Clinical Diagnostics Laboratory for measurement of p‐tau217^8^; and to appropriate laboratories for genetic studies including genome and ribonucleic acid (RNA) sequencing and proteomics. Blood‐based biomarker results not presented here or analyzed by other assays will be presented separately. For the present analysis, the Aβ40 and Aβ42 values and the apoE results are from C_2_N, the t‐tau and p‐tau181 data are from Quanterix, and the p‐tau217 data are from Lilly Clinical Diagnostics Laboratory.

Only the blood assay results of the lipid panel were returned to the PI for review to determine any clinically significant abnormal laboratory findings and/or the need for participant follow‐up to address non–study‐related health issues.

### 2.5 Brain amyloid PET scan

Brain amyloid PET scans were conducted at a designated imaging facility near each site. Prior to the approximate 20‐minute scan, the US Food and Drug Administration (FDA)–approved tracer, Amyvid, florbetapir F‐18 (18F‐AV‐45), was injected intravenously with a target per the label dosage and administration instructions.^29^ To improve consistency of amyloid PET scan interpretations, all locally developed images were uploaded into an electronic imaging portal that were accessible by IXICO's network of specialists trained to interpret brain florbetapir PET images. Hence, all brain amyloid PET scans were centrally read by an expert trained in the manufacturer read process and following a process in which the reader had visibility to a subject's standardized uptake value ratio (SUVR) value but made the final determination according to manufacturer standards. Scan results were disseminated to each research site. Results of the scans were discussed with the participants at Visit 3 by a trained medical professional.

### 2.6 CSF collection

One research site centrally serving and recruiting from a largely Hispanic population in South Texas did not have local access to a qualified PET imaging center. For participants at this site a CSF sample was collected and analyzed for Aβ40, Aβ42, and Aβ42/Aβ40 by Quest Diagnostics, a CLIA‐certified laboratory.

The CSF sample was acquired via a lumbar puncture performed by a qualified physician. Prior to the procedure, a coagulation panel and computerized tomography (CT) scan of the brain were reviewed to rule out any contraindications. CSF sampling was performed between 8:00 am and 12:00 pm to minimize diurnal variation of CSF parameters. CSF samples were sent to Quest Diagnostics’ CLIA central lab for analysis and the results were disseminated to the site. The results of the CSF analysis were discussed with the participants at Visit 3 by a trained medical professional.

### 2.7 Statistical analysis

#### 2.7.1 General statistical considerations

The primary purpose of this study is to determine the relationship of blood and digital biomarker measurements to the presence of amyloid plaques in the brain identified through brain amyloid PET or CSF analysis. (Digital biomarker data will be presented in future publications.) The relationship of clinical and demographic variables and potential blood‐based biomarkers to brain amyloid was determined for participants in the three study cohorts (CN, MCI, and mild AD). Because studies show the rate of amyloid PET positivity in persons > age 65 with normal cognition are usually 10% to 30%,^30^ recruitment monitoring slightly oversampled the CN cohort to increase the number of amyloid PET–positive cases. Recruitment monitoring also ensured ≥ 20% representation of URP across the three cohorts of CN, MCI, and AD. Statistical analyses were performed by Pentara Corporation using SAS version 9.4 or R version 4.2.2 or more recent statistical software.

To initially characterize the relationship between clinical and demographic variables and amyloid status, both *t* tests and analysis of covariance (ANCOVA) tests were performed for all continuous variables. Additionally, *t* tests were used to assess differences in individual blood biomarkers between participants with and without brain amyloid plaques. For the blood‐based biomarkers showing the greatest differences between amyloid‐positive and amyloid‐negative participants receiver operating characteristic (ROC) analyses were performed. No adjustments for multiple comparisons were made and only differences with nominal significance of at least *P* < 0.01 are noted in the results discussion. For ROC curves, area under the ROC curves (AUCs) were also calculated and adjusted values were used to assess the impact of age and apoE ε4 status (carrier vs. non‐carrier) because of the strong relationship of age and apoE ε4 status to amyloid positivity. The ability of blood‐based biomarkers to predict amyloid positivity was assessed using logistic regression analyses as implemented in SAS proc logistic. ROC curves and associated AUC were calculated using the pROC package and proc logistic in R and SAS, respectively. Differences in AUCs were compared using the method of DeLong et al.^31^ Logistic regression was performed with amyloid PET as the outcome or dependent variable and biomarkers as the independent or predictor variable. A secondary logistic regression analysis was performed with age and apoE ε4 status (carrier/non‐carrier) as covariates. Subgroup analyses by ethnicity were also performed with and without covariate adjustment. For categorical variables (clinical cohort, sex, and apoE ε4 carrier status), chi square or Cochran–Mantel–Haenszel statistics were used to evaluate the relationship to brain amyloid status. Due to the Bio‐Hermes Study's robust database, it is anticipated that additional statistical analysis will be conducted by study partners, and with appropriate other partners with expertise in artificial intelligence and/or machine learning to possibly identify a predictive multivariate biomarker relationship or algorithm to improve prediction capabilities of amyloid PET.

#### 2.7.2 Secondary analyses

Exploratory analyses were performed to identify any potential differences related to URP in biomarker thresholds and their relationship to brain amyloid positivity status. Differences in biomarker thresholds between groups defined by race and ethnicity were evaluated with logistic regression and AUC standard errors were calculated as described above. Some participants had biomarker values that were below the lower limit of quantitation (<LLOQ) or the lower limit of detection (<LOD) for the assay used. This was most common for measures of p‐tau181 and p‐tau217, for which 100 and 169 participants, respectively, had values below the LLOQ or LOD. In those cases, a random value was imputed between zero and the LLOQ or LOD because those participants were known to have very low values. There were seven values above the upper limit of quantitation (ULOQ) for p‐tau217. In those cases, a value equal to the ULOQ was assigned. AUCs are reported both without imputation and with imputed values and both with and without adjustment for age and apoE ε4.

## 3 RESULTS

### 3.1 Participant characteristics (total population)

Table 1 presents the demographic variables, clinical measures, and apoE ε4 results for each of the clinical cohorts separated by brain amyloid status along with results for the total Bio‐Hermes Study population. Across the three clinical cohorts the rate of amyloid positivity increased with greater cognitive impairment, from 21% (84 of 400) for CN participants to 34.7% (103 of 297) for participants with MCI to 60.2% (156 of 259) in the mild AD group (*P* < 0.001). For the total population enrolled (*N* = 1001), brain amyloid‐positive participants were older (*P* < 0.0001) than amyloid‐negative participants; the amyloid relationship with older age was clear in the CN group and the MCI group and marginal in the mild AD group (*P* < 0.0001 for CN, *P* = 0.0007 for MCI, *P* = 0.0490 for mild AD groups). Overall mean age (years) was in the early 70s similar to the mean age of participants enrolled in most therapeutic research studies.^32^ There was no significant association of sex with amyloid status in the population as a whole or in any of the clinical groups. Education level was also not related to amyloid status for the group as a whole or for any of the clinical groups. The proportion of more females than males in the Bio‐Hermes Study is also similar to that observed in most therapeutic trials.^33^ Education levels were generally high and comparable to those observed in many therapeutic trials.^19^ Cognitive and functional measures were generally worse in participants who were amyloid positive compared to those who were amyloid negative across the total study population (*P* < 0.0001 for MMSE, RAVLT, and FAQ). The differences for the MMSE and RAVLT related to amyloid status were greater in the mild AD group than in either the CN or the MCI group. The FAQ scores were slightly but significantly higher (more impaired) among amyloid‐positive participants in the CN (*P* = 0.0083) and MCI (*P* < 0.0001) but not in the mild AD (*P* = 0.4864) groups. A strong relationship of apoE ε4 status to amyloid positivity was observed overall and in each of the clinical groups; in all groups the prevalence of apoE ε4 carriers was significantly higher among amyloid‐positive than among amyloid‐negative participants (*P* < 0.0001 in each case).

**TABLE 1:** Population general demographics by clinical status.

	Cognitive normal (CN)			Mild cognitive impairment (MCI)			Mild Alzheimer's disease			Total population		
General demographics	Amyloid negative (*N* = 316)	Amyloid positive (*N* = 84)	*P* value	Amyloid negative (*N* = 194)	Amyloid positive (*N* = 103)	*P* value	Amyloid negative (*N* = 103)	Amyloid positive (*N* = 156)	*P* value	Amyloid negative (*N* = 613)	Amyloid positive (*N* = 343)	*P* value
Age, mean (SD)	69.7 (6.23)	72.9 (6.46)	<0.0001	71.2 (6.96)	74.0 (6.19)	0.0007	73.5 (6.67)	75.0 (5.78)	0.0490	70.8 (6.67)	74.2 (6.12)	<0.0001
Female/male, *n/n*	196/120	47/37	0.311	104/90	56/47	0.9004	57/46	80/76	0.522	357/256	183/160	0.1439
Education, yrs, mean (range)	15.8 (8.0, 24.0)	15.6 (12.0, 23.0)	0.5571	15.3 (2.0, 23.0)	15.7 (9.0, 22.0)	0.2550	14.9 (6.0, 20.0)	14.8 (4.0, 24.0)	0.8475	15.5 (2.0, 24.0)	15.3 (4.0, 24.0)	0.2421
MMSE, mean (SD)	28.4 (1.49)	28.5 (1.36)	0.7213	27.4 (1.94)	26.8 (1.94)	0.0267	23.9 (2.40)	22.7 (2.54)	0.0002	27.3 (2.42)	25.4 (3.27)	<0.0001
RAVLT, mean (SD)	47.9 (13.37)	46.8 (13.63)	0.5076	37.5 (12.28)	37.1 (10.68)	0.7840	32.3 (13.79)	30.0 (11.01)	0.1318	42.0 (14.53)	36.2 (13.39)	<0.0001
FAQ, mean (SD)	0.6 (1.37)	1.1 (1.86)	0.0083	2.9 (3.77)	5.0 (5.14)	<0.0001	9.1 (7.70)	9.7 (6.20)	.4864	2.7 (4.94)	6.2 (6.20)	<0.0001
ApoE ε4 carrier/non‐carrier, *n/n*	82/233	48/36	<0.0001	46/145	67/36	<0.0001	12/89	98/55	<0.0001	140467	213/127	<0.0001
Non‐Hispanic Whites, *n*	252	72	–	144	83	–	60	116	–	456	271	–
Hispanic, *n*	27	1	–	22	13	–	21	25	–	70	39	–
Non‐Hispanic Blacks, *n*	32	10	–	23	6	–	21	11	–	76	27	–
Other, *n*	5	1	–	5	1	–	1	4	–	11	6	–

*Notes*: *P* values for age, education, MMSE, RAVLT, and FAQ were generated using a *t* test; *P* values for sex and apoE ε4 status were generated using a chi‐square test. *P* values not calculated for individual ethnoracial groups due to small populations.

Abbreviations: ApoE, apolipoprotein E; FAQ, Functional Activities Questionnaire; MMSE, Mini‐Mental State Examination; RAVLT, Rey Auditory Verbal Learning Test; SD, standard deviation.

In summary, these results show strong relationships of amyloid positivity with older age and with a higher prevalence of apoE ε4 carriers across all clinical groups.

### 3.2 Blood‐based biomarkers and brain amyloid positivity

Table 2 presents the blood‐based biomarker results for each of the clinical groups and for the total Bio‐Hermes Study population. These data show no significant relationship of Aβ40 or t‐tau with amyloid positivity. Each of the other biomarkers shows a significant relationship with amyloid positivity in each of the clinical groups. For Aβ42, lower values were found in the amyloid‐positive participants across all groups (*P* = 0.0009 for CN and *P* < 0.0001 for both MCI and mild AD groups). P‐tau181 (higher values in the amyloid‐positive participants, *P* < 0.0001 in all groups) and p‐tau217 (higher values in the amyloid‐positive participants, *P* < 0.0001 in all groups) showed strong relationships with amyloid positivity. These findings are broadly consistent with previous results showing significant relationships of Aβ42/Aβ40, p‐tau181, and p‐tau217 with amyloid positivity.^34^, ^35^

**TABLE 2:** Clinical status and blood‐based biomarkers.

	Cognitive normal (CN)			Mild cognitive impairment (MCI)			Mild Alzheimer's disease			Total population		
Blood‐based biomarker	Amyloid negative (*N* = 316)	Amyloid positive (*N* = 84)	*P* value	Amyloid negative (*N* = 194)	Amyloid positive (*N* = 103)	*P* value	Amyloid negative (*N* = 103)	Amyloid positive (*N* = 156)	*P* value	Amyloid negative (*N* = 613)	Amyloid positive (*N* = 343)	*P* value
Aβ40 pg/mL, mean (SD)	494.4544 (93.7974)	504.4041 (102.7125)	0.3980	507.3705 (99.0066)	512.5435 (91.7351)	0.6617	495.2180 (96.2563)	498.1427 (103.4304)	0.8214	498.6438 (95.8925)	504.0523 (99.7468)	0.4125
Aβ42 pg/mL, mean (SD)	49.4778 (9.4999)	45.6459 (8.4988)	0.0009	50.5300 (9.4631)	45.7033 (8.3810)	<0.0001	50.2842 (9.5715)	45.5334 (8.9748)	<0.0001	49.9423 (9.4971)	45.6127 (8.6568)	<0.0001
Aβ42/Aβ40, mean (SD)	0.1004 (0.0089)	0.0911 (0.0087)	<0.0001	0.1000 (0.0096)	0.0893 (0.0060)	<0.0001	0.1020 (0.0091)	0.0920 (0.0080)	<0.0001	0.1006 (0.0092)	0.0909 (0.0077)	<0.0001
P‐tau181 pg/mL, mean (SD)	15.3654 (10.6974)	22.3410 (8.9829)	<0.0001	16.2528 (11.3931)	24.6190 (9.8849)	<0.0001	16.5356 (11.6583)	28.0207 (19.0003)	<0.0001	15.8394 (11.0757)	25.5798 (14.7020)	<0.0001
P‐tau217 U/mL, mean (SD)	0.1442 (0.0763)	0.2684 (0.1084)	<0.0001	0.1576 (0.1189)	0.3698 (0.2355)	<0.0001	0.2001 (0.2100)	0.5062 (0.3061)	<0.0001	0.1577 (0.1230)	0.4065 (0.2670)	<0.0001
T‐tau pg/mL, mean (SD)	2.1929 (2.1816)	2.1667 (0.9786)	0.9147	2.7317 (6.7227)	2.0149 (0.8765)	0.2827	2.6735 (6.8558)	2.0485 (1.0845)	0.2720	2.4419 (4.9420)	2.0677 (0.9977)	0.1691

*Notes*: Values indicated to be below the lower limit of quantitation were assigned an imputed value between 0 and the lower limit of quantitation. Values above the upper limit of quantitation were assigned the value at the upper limit of quantitation. *P* values were generated using a standard *t* test.

Abbreviations: Aβ, amyloid beta; p‐tau, phosphorylated tau; SD, standard deviation; t‐tau, total tau.

### 3.3 Participant demographics (underrepresented populations)

Consistent with Figure 1, the URP population was represented in each of the clinical groups with 80, 76, and 90 URP participants among the CN, MCI, and mild AD cohorts, respectively. Table 3 presents additional demographic and clinical data for Bio‐Hermes participants separated by race and ethnicity. The URP excludes the non‐Hispanic White population (*n* = 775); to avoid duplication, the Hispanic group includes those who identified as Hispanic or Latino (*n* = 115) and the non‐Hispanic Black group includes those who identified as Black or African American (*n* = 112). The Other URP group is small (*n* = 19) and included participants who identified as being Asian, American Indian, or Alaska native.

**TABLE 3:** Ethnicity and race demographics.

General demographics	Non‐Hispanic White (*N* = 755)	Hispanic (*N* = 115)	*P* value	Non‐Hispanic Black (*N* = 112)	*P* value	Other (*N* = 19)
Age, mean (SD)	72.5 (6.62)	71.1 (6.93)	0.0451	70.2 (6.21)	0.0008	71.0 (7.38)
Female/male, *n/n*	407/348	66/49	0.4847	80/32	0.0005	10/9
Education, yrs, mean (range)	15.6 (8.0, 24.0)	14.6 (4.0, 23.0)	0.0002	14.7 (2.0, 21.0)	0.0005	17.5 (9.0, 20.0)
MMSE, mean (SD)	27.0 (2.75)	25.3 (3.24)	<0.0001	25.3 (2.80)	<0.0001	25.5 (3.58)
FAQ, mean (SD)	3.8 (5.44)	5.3 (6.68)	0.0100	3.9 (6.50)	0.9829	3.8 (5.54)
ApoE ε4 carrier/non‐carrier, *n/n*	285/461	37/77	0.2376	42/69	0.941	6/13
Amyloid pos/total n for PET or CSF (%)	271/727 (37.3%)	39/109 (35.8%)	0.0778	27/103 (26.2%)	0.0014	6/17 (35.3%)

*Notes*: *P* values for age, education, MMSE, and FAQ for individuals not in the non‐Hispanic White populations were generated using a standard *t* test vs. the non‐Hispanic White population; *P* values for sex and apoE status were generated using a chi‐square test. *P* values for amyloid positivity rates were generated by logistic regression using age, sex, and education as covariates. *P* values were not calculated for the other cohort due to the small population.

Abbreviations: ApoE, apolipoprotein E; CSF, cerebrospinal fluid; FAQ, Functional Activities Questionnaire; MMSE, Mini‐Mental State Examination; PET, brain positron emission tomography; SD, standard deviation.

Because the non‐Hispanic White group is most often included in clinical trials, this article will focus on analyses to determine possible differences between the larger URP groups of Hispanic and non‐Hispanic Black compared to non‐Hispanic White participants. The proportion of participants who were positive for brain amyloid by either PET or CSF was lower for non‐Hispanic Blacks (26.2%; *P* = 0.0014) compared to non‐Hispanic Whites (37.3%); the proportion for Hispanics (35.8%) did not differ from that for non‐Hispanic Whites (*P* = 0.0778). Both Hispanic (mean difference of 1.4 years, *P* = 0.0451) and non‐Hispanic Black (mean difference of 2.3 years, *P* = 0.0008) participants tended to be younger than non‐Hispanic White participants. The proportion of females to males was similar for non‐Hispanic White and Hispanic groups but there was a lower percentage of males among non‐Hispanic Black relative to non‐Hispanic White participants (*P* = 0.0005). Both Hispanic and non‐Hispanic Black participants had slightly, but significantly, fewer years of education (*P* = 0.0002, *P =* 0.0005, respectively) than did non‐Hispanic White participants; all groups had education levels with a mean of > 2 years of postsecondary education. This is similar to the education level found in most clinical trial participants.^3^, ^19^ Cognitive scores were generally lower among the ethnic minority groups than among non‐Hispanic Whites while FAQ scores were higher (more impaired) among Hispanic but not among the non‐Hispanic Black participants relative to non‐Hispanic White participants. Importantly, there were no significant differences between race and ethnic groups in the proportion of participants carrying apoE ε4 compared to non‐Hispanic Whites (*P* = 0.2376 for Hispanic and *P* = 0.941 for non‐Hispanic Black groups). While the demographic differences among groups by race and ethnicity are not large, they do indicate that the groups should at least be considered separately in the prescreening for amyloid status.

### 3.4 Biomarker concentration and ethnic and racial cohorts

Table 4 presents the key blood‐based biomarker mean concentrations and SD for the participants grouped by race and ethnicity. The average p‐tau181 (*P* = 0.0006) and p‐tau217 (*P* = 0.0003) values were significantly lower among non‐Hispanic Black participants compared to non‐Hispanic Whites; these differences were confirmed by ANCOVA analyses using sex, age, education, and baseline MMSE as covariates (*P* < 0.0001 in both cases). The average p‐tau181 and p‐tau217 values among Hispanics did not differ significantly from those for non‐Hispanic White participants (*P* = 0.0661 and *P* = 0.3132, respectively) by *t* test but the difference for p‐tau 181 was significant after adjusting for ethnicity, baseline MMSE, age, sex, and education status (*P* = 0.004). The average Aβ42/Aβ40 ratio was higher in both Hispanic (*P* = 0.0026) and non‐Hispanic Black (*P* < 0.0001) participants compared to the non‐Hispanic White group. Overall, the average concentration of all three biomarkers reported for non‐Hispanic Black participants was significantly less (*P* < 0.001) than concentration values reported for non‐Hispanic White participants.

**TABLE 4:** Ethnicity and race and select blood‐based biomarkers.

Blood‐based biomarker	Non‐Hispanic White	Hispanic	*P* value	Non‐Hispanic Black	*P* value	Other races
P‐tau181 pg/mL, mean (SD)	20.1271 (14.0411)	17.5958 (11.0046)	0.0661	15.4135 (8.1258)	0.0006	17.5873 (11.8316)
P‐tau217 U/mL, mean (SD)	0.2528 (0.2281)	0.2764 (0.2614)	0.3132	0.1730 (0.1144)	0.0003	0.2777 (0.2611)
Aβ42/Aβ40, mean (SD)	0.0960 (0.0094)	0.0990 (0.0110)		0.1021 (0.0104)		0.0968 (0.0068)

*Notes*: Values indicated to be below the lower limit of quantitation were assigned an imputed value between 0 and the lower limit of quantitation. Values above the upper limit of quantitation were assigned the value at the upper limit of quantitation. *P* values were generated using a standard *t* test.

Abbreviations: Aβ, amyloid beta; p‐tau, phosphorylated tau; SD, standard deviation.

Table 5 presents the AUC values for each of the three biomarkers for the total population and separately for participants in the three principal groups by ethnicity and race. AUC values are shown without imputed and with imputed values and as unadjusted and adjusted for age and apoE ε4 carrier status. For the entire population these adjustments by logistical regression increased the AUC for Aβ42/Aβ40 ratio (from 0.8032 to 0.8463).

**TABLE 5:** AUC values across populations with no imputation and imputation values and unadjusted and adjusted for age and apoE ε4.

	No imputation			Imputation		
Parameter	*N*	AUC unadjusted (SE)	AUC adjusted (SE)	*N*	AUC unadjusted (SE)	AUC adjusted (SE)
**Total population**						
P‐tau181	846	0.7739 (0.0161)	0.8230 (0.0144)	946	0.7758 (0.0158)	0.8316 (0.0135)
P‐tau217	777	0.8885 (0.0124)	0.8885 (0.0119)	946	0.9067 (0.0104)	0.9087 (0.0097)
Aβ42/Aβ40	944	0.8032 (0.0149)	0.8463 (0.0128)	944	0.8032 (0.0149)	0.8463 (0.0128)
**Non‐Hispanic White**						
P‐tau181	657	0.7785 (0.0181)	0.8279 (0.0162)	719	0.7633 (0.0187)	0.8284 (0.0156)
P‐tau217	602	0.8955 (0.0136)	0.8892 (0.0135)	719	0.9087 (0.0119)	0.9046 (0.0114)
Aβ42/Aβ40	717	0.8077 (0.0167)	0.8557 (0.0143)	717	0.8077 (0.0167)	0.8557 (0.0143)
**Hispanic**						
P‐tau181	87	0.7911 (0.0493)	0.8260 (0.0438)	108	0.8538 (0.0364)	0.8752 (0.0331)
P‐tau217	85	0.8733 (0.0382)	0.8719 (0.0386)	108	0.8964 (0.0317)	0.9064 (0.0283)
Aβ42/Aβ40	108	0.7910 (0.0458)	0.8120 (0.0426)	108	0.7910 (0.0458)	0.8120 (0.0426)
**Non‐Hispanic Black**						
P‐tau181	87	0.7117 (0.0571)	0.8346 (0.0438)	102	0.7694 (0.0476)	0.8578 (0.0383)
P‐tau217	73	0.8128 (0.0564)	0.8768 (0.0407)	102	0.8852 (0.0361)	0.9240 (0.0260)
Aβ42/Aβ40	102	0.7583 (0.0546)	0.8652 (0.0382)	102	0.7583 (0.0546)	0.8652 (0.0382)

*Notes*: *N* values reflect lab assays available for analyses. Values indicated to be below the lower limit of quantitation were assigned an imputed value between 0 and the lower limit of quantitation. Values above the upper limit of quantitation were assigned the value at the upper limit of quantitation. *P* values were generated using logistic regression as implemented in SAS proc logistic.

Abbreviations: Aβ, amyloid beta; apoE, apolipoprotein E; AUC, area under the receiver operating characteristic curve; p‐tau; phosphorylated tau; SE, standard error.

For the total population, including imputed values increased the AUC for p‐tau181 (from 0.8230 to 0.8316 for adjusted values; from 0.7739 to 0.7758 for unadjusted values) and increased the AUC for p‐tau217 (from 0.8885 to 0.9087 for adjusted values and from 0.8885 to 0.9067 for unadjusted values). There were no marked differences in AUCs between the different ethnic and racial groups. The adjustment and imputation resulted in slightly higher AUC values for all groups except for the non‐Hispanic White population in which the imputed and unadjusted value for p‐tau217 was minimally higher compared to imputed adjusted value (0.9087 vs. 0.9046); for all groups, the AUC for p‐tau217 was largest.

**FIGURE 2:**
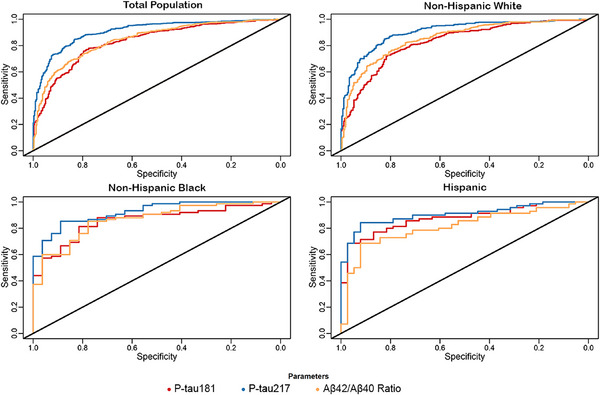
ROC curves for prediction of brain PET or CSF amyloid positivity adjusted for age and apoE ε4 status with imputed values. Aβ, amyloid beta; apoE, apolipoprotein E; CSF, cerebrospinal fluid; PET, positron emission tomography; p‐tau, phosphorylated tau; ROC, receiver operating characteristic.

Figure 2 presents the ROC curves for the three most promising biomarkers: p‐tau181, p‐tau217, and Aβ42/Aβ40. ROC curves are provided for the Bio‐Hermes population as a whole and for the three race and ethnic groups separately. For the total population the ROC curve for p‐tau 217 was significantly better at predicting amyloid status than those for Aβ42/Aβ40 and p‐tau 181 (*P* < 0.0001) but the latter two groups were not different (*P* = 0.18). Because all the biomarkers’ AUCs using adjusted and imputed values were > 0.8000, and in most instances > 0.8500, ROC curve results used AUC data adjusted for age and apoE ε4 status and imputed values are included for p‐tau181 and p‐tau217. The following are the number of imputed values for each group, total population (*n* = 269), non‐Hispanic White (*n* = 179), Hispanic (*n* = 44), and non‐Hispanic Black (*n* = 44). The specific values for sensitivity and specificity depend upon the cutoff chosen and optimal cutoffs will vary depending upon tolerance for false positives and false negatives. For the total population, and for each of the subgroups, the ROC curve for p‐tau217 was above those for the other two biomarkers but all have ROC curves sufficient to provide good predictive power for brain amyloid status.

## 4 DISCUSSION

The Bio‐Hermes Study was designed to provide a platform dataset to enable evaluation of blood‐based and digital biomarkers for their ability to identify persons who are brain amyloid positive in a racially and ethnically diverse group of persons screened at clinical trial recruiting sites. The aim of the current analysis was to evaluate widely used blood‐based biomarkers in predicting brain amyloid positivity. Results indicate that Aβ42/Aβ40 ratio, p‐tau181, and p‐tau217 are good predictors of brain amyloid positivity in this clinical trial–ready population and suggest that further separate evaluation of biomarkers for Hispanic or non‐Hispanic Black participants may be useful.

Of these promising biomarkers, p‐tau217 was numerically superior for the entire population and for each race and ethnic group examined separately; however, all showed ROC curves adjusted for age and apoE ε4 (with no imputation) with AUCs ≥ 0.8120 relative to brain amyloid positivity. Some values of both plasma p‐tau181 and p‐tau217 were below the LLOQ, and some p‐tau217 values were greater than the ULOQ; using a simple imputation scheme improved the AUCs for both measures and suggests that such an imputation scheme will enable the use of these measures for prediction of brain amyloid even when values are below the LLOQ (or above ULOQ). Addition of age and apoE ε4 carrier status as variables with imputed values improved the AUCs somewhat for p‐tau181 and for p‐tau217 suggesting that this additional information is a useful addition when constructing ROC curves for these variables.

The demographic characteristics of the participants enrolled in the Bio‐Hermes studies are similar to those screened for recent AD clinical trials.^36^, ^37^ As is the case with therapeutic trials, the rate of brain amyloid positivity among the clinical cohorts increased with increasing cognitive impairment. Regardless of cognitive status, brain amyloid positivity was associated with older age and the presence of apoE ε4 but was not associated with sex or education. The associations observed in the Bio‐Hermes data show AUC values similar to, or even larger than, those from other biomarker validation studies.^8^, ^9^, ^38^ We found small, but significant, differences in the average age, average education level, and proportion of females to males between racial and ethnic groups. Non‐Hispanic Whites tended to be slightly older and had more years of education than either Hispanic or non‐Hispanic Black participants; additionally, the percentage of females in the non‐Hispanic Black participants was higher than in either the non‐Hispanic White or the Hispanic participants. These differences reflect to some extent differences in the US population as a whole, in which non‐Hispanic Blacks and Hispanics in the age of highest risk for AD (> 65) tend to be younger and have fewer years of education than non‐Hispanic Whites.^20^, ^39^, ^40^ We also found that the average values for Aβ42/Aβ40 were slightly higher in non‐Hispanic Blacks and Hispanics than in non‐Hispanic Whites and both p‐tau181 and p‐tau217 concentrations were lower on average among non‐Hispanic Blacks than non‐Hispanic Whites. Among Hispanic participants, the p‐tau181 and p‐tau217 average values were not significantly different relative to non‐Hispanic Whites. One previous study looking at CSF biomarkers found that average p‐tau 181 values were significantly higher among non‐Hispanic Whites than among non‐Hispanic Blacks.^7^

The ability of blood‐based biomarkers to predict amyloid positivity was quite similar across the three race/ethnicity groups. The group differences in demographic characteristics and average biomarker values did not have any significant effect on the predictive power of Aβ42/40, p‐tau181, or p‐tau217 for amyloid positivity. Another biomarker study, using an autopsy cohort, evaluated White/non‐Hispanic, Hispanic, and African American cohorts, with mean age ≈ 10 years older than the Bio‐Hermes Study participants found AUC values lower than those obtained in Bio‐Hermes Study, but the values were similar for the different race/ethnicity groups.^38^ However, a study comparing blood‐based biomarkers for their ability to predict CSF Aβ42/Aβ40 results found plasma Aβ42/Aβ40 a more consistent predictor than p‐tau181, p‐tau231, or NfL among non‐Hispanic Blacks.^41^ The potential impact of demographic differences by race and ethnicity on screen failure rates in clinical trial recruitment is not entirely clear. One possibility is that a larger number of Hispanic and non‐Hispanic Black persons may need to be screened to enroll a representative proportion of clinical trial participants who have amyloid deposits. It is also possible that a different threshold for brain amyloid positivity would decrease the screen failure rate among members of URPs. The impact of any change in brain amyloid threshold for enrollment on clinical efficacy would need to be assessed in clinical trials.

In summary, the Bio‐Hermes analyses of the promising blood‐based biomarkers for the three race and ethnicity groups showed that p‐tau181, Aβ42/Aβ40, and particularly p‐tau 217 biomarkers were significant predictors of amyloid positivity in all three groups. The Bio‐Hermes Study provides useful data to select prescreening blood‐based biomarkers across diverse ethnic, racial, and clinical cohorts to facilitate the assessment of those likely to be brain amyloid positive. Enhancing the prescreen process with blood‐based assays could ultimately reduce the number of potential clinical trial participants who screen fail due to negative brain amyloid results obtained through more invasive or costly procedures.

The Bio‐Hermes Study has limitations and offers opportunities for future research. Among the limitations is the use of conventional cognitive tests to assign participants to clinical groups regardless of ethnicity and race. Tests such as the MMSE and RAVLT may have biases making them less effective in members of underrepresented minority groups. Also, while we were able to recruit larger numbers of minority participants than is usually the case in clinical trials, the number of Hispanic and non‐Hispanic Black participants was still small compared to the number of non‐Hispanic Whites. Last, we have only analyzed some of the blood and digital data obtained in Bio‐Hermes Study with the major blood biomarker results reported here. Blood samples were also collected for measurement of biomarkers such as NfL, GFAP, and others that may facilitate prediction of cognitive state and amyloid status. More granular cognitive measures were also obtained from some participants. We anticipate publications resulting from analyses of the entire Bio‐Hermes Study database in the future.

ClinicalTrials.gov ID: NCT04733989, A Biomarker Database to Investigate Blood‐Based and Digital Biomarkers in Participants Screened for Alzheimer's Disease (Bio‐Hermes). Sponsor, GAP Innovations, PBC.

## CONFLICT OF INTEREST STATEMENT

The following reviewed and edited the manuscript for accuracy: Richard C. Mohs, Douglas Beauregard, John Dwyer, Jennifer Gaudioso, Jason Bork, Tamiko MaGee Rodgers, Mickeal Key, Lynn Hughes, and Cyndy Cordell are employees/advisors of the Global Alzheimer's Platform Foundation. Lynn Hughes is also an advisor to IXICO PLC. Diana R. Kerwin is Founder and President of Kerwin Medical Center, Dallas, TX. Jessie Nicodemus‐Johnson and Craig Mallinckrodt are employees of Pentara Corporation. Robin Wolz is an employee of IXICO PLC. Kevin Yarasheski, Joel B. Braunstein, Tim West, Philip Verghese, Kris Kirmess, and Matthew Meyer are employees of C2N Diagnostics. David Wilson is an employee of Quanterix, and experts employed by Eli Lilly and Company. Author disclosures are available in the supporting information.

## CONSENT STATEMENT

Prior to the start of any study procedures, an informed consent was obtained from all study participants.

## Supporting information

Supporting Information

## 1

Collaborators:

Jessie Nicodemus‐Johnson^1^, Craig Mallinckrodt^1^, Robin Wolz^2^, Kevin Yarasheski^3^, Joel B. Braunstein^3^, Tim West^3^, Philip Verghese^3^, Kris Kirmess^3^, Matthew Meyer^3^, David Wilson^4^

^1^Pentara Corporation, Millcreek, Utah, USA.

^2^IXICO PLC, London, UK.

^3^C2N Diagnostics, St. Louis, Missouri, USA.

^4^Quanterix, Billerica, Massachusetts, USA.
